# Implementation and Evaluation of a Virtual Elective in Otolaryngology in the Time of COVID-19

**DOI:** 10.1177/0194599820951150

**Published:** 2020-08-11

**Authors:** Andrew J. Steehler, Brian Pettitt-Schieber, Matthew B. Studer, Geetha Mahendran, Barbara J. Pettitt, Oswaldo A. Henriquez

**Affiliations:** 1 Department of Surgery, School of Medicine, Emory University, Atlanta, Georgia, USA; 2 Department of Otolaryngology–Head and Neck Surgery, School of Medicine, Emory University, Atlanta, Georgia, USA

**Keywords:** otolaryngology, COVID-19, remote education, medical education

## Abstract

**Objective:**

To develop and evaluate a virtual otolaryngology medical student elective created during the COVID-19 crisis with the intention of teaching the basic tenets of otolaryngology and increasing exposure to the specialty.

**Study Design:**

Cross-sectional survey.

**Setting:**

Emory University School of Medicine.

**Methods:**

A 1-week virtual otolaryngology curriculum was offered to third- and fourth-year medical students that centered on the American Academy of Otolaryngology–Head and Neck Surgery Foundation’s handbook *Primary Care in Otolaryngology* (fourth edition). The course covered a variety of topics and was conducted remotely via online video conferencing software. We applied multiple teaching modalities and surveyed students regarding the effectiveness of the course. Mixed methods analysis was employed to analyze the course data.

**Results:**

Twelve students participated; 67% reported their baseline precourse understanding of otolaryngology in the “poor-fair” range. After the course, 92% of students reported increased understanding, with 42% and 58% reporting “good” and “very good” understanding, respectively. Following completion of the course, posttest scores on summative assessments were significantly higher than pretest scores (*P* < .001). Ninety-two percent of students reported either “increased” or “greatly increased” interest in otolaryngology postcourse. Qualitative survey results revealed students’ appreciation of course organization, formative assessments, and case-based learning.

**Conclusions:**

An otolaryngology elective administered through a virtual format can be effective at providing an educational experience and garnering interest in the field. Positive exposure to otolaryngology can increase medical students’ interest in pursuing the specialty and expand their general knowledge of consultation, diagnosis, and management in otolaryngology.

Clinical experiences are vital to the medical student learning process. Among third-year medical student electives, surgery demands hands-on participation. Due to the COVID-19 outbreak, the Association of American Medical Colleges recommended that medical schools across the country suspend clinical rotations through March 31, a recommendation that was later extended to April 14.^1^ This implementation primarily affected third- and fourth-year medical students whose curriculum revolves around clinical learning. As such, another consequence of the COVID-19 pandemic was a decrease in medical student clinical exposure to all medical specialties, including otolaryngology, a field already neglected in most medical school curricula.^2^

During this clinical clerkship pause, the Association of American Medical Colleges suggested that medical education communities develop appropriate educational strategies and alternatives to fill in these crucial clinical experience gaps.^1^ In particular, the creation of a remote-learning, multi-institutional, open-access curriculum for the otolaryngology-interested medical student has been suggested.^3^ During April-May 2020, faculty, residents, and senior medical students at our institution created a virtual 1-week otolaryngology elective intended to serve as a blueprint for these efforts. Our objective was to provide third- and fourth-year medical students of all interests with valuable education and insight to the field, encouraging students to consider otolaryngology as a possible career and creating an online archive of our work that will be accessible for future classes. Here, we describe the development, content, and teaching modalities of our curriculum, as well as the data demonstrating the effectiveness of the course in achieving its objectives.

## Methods

### Course Development

Development of the virtual otolaryngology elective began 4 weeks in advance of the first course offering. In conjunction with faculty and residents at Emory University, senior medical students identified the priorities and objectives of the course. Understanding that the course would be offered to third- and fourth-year medical students of varying backgrounds, interests, and experience with surgical fields and otolaryngology, we chose to prioritize teaching the foundations of otolaryngology that are important for physicians in any specialty. These foundational concepts would help students learn the pathophysiology, workup, and treatment of otolaryngologic diseases, as well as when to order a referral. To that end, the American Academy of Otolaryngology–Head and Neck Surgery Foundation’s (AAO-HNSF’s) publication *Primary Care Otolaryngology* (fourth edition) was chosen as the required reading material for the course.^4^ This handbook is designed to be a concise, comprehensive introduction to the field of otolaryngology. It facilitates the utility of our course by discussing patient complaints that may be handled safely via primary care providers, pediatricians, and any other specialty that would benefit from a foundational knowledge of otolaryngology.

The development of learning goals and objectives for the course arose from the learning objectives of the optional third-year clinical otolaryngology elective and the third-year general surgery clerkship:

1. Students will be introduced to the head and neck examination and the breadth of clinical problems seen by otolaryngologists.
2. Students will use critical thinking skills to develop a differential diagnosis and learn the management of common otolaryngology presenting symptoms.
3. Students will learn when it is appropriate to call for otolaryngology consultation.
4. Students will demonstrate their understanding of common otolaryngology topics in the form of a 5-minute presentation and receive feedback.
5. Students will learn how various patient factors and medical illnesses may lead to and/or affect the course of otolaryngologic disease.
6. Students will become acquainted with otolaryngologists and their practices.

#### Curriculum

The curriculum consisted of 4 days of didactic sessions with faculty and residents in the Department of Otolaryngology, delivered by commercially available online video conferencing software (Zoom). Prior to the start of the course, students were asked to watch 4 short ear, nose, and throat examination videos found on the AAO-HNSF website, lasting 36 minutes in total. These videos covered anatomy and examination of the ear, oral cavity and neck, face and nose, and nasopharynx and larynx with common abnormalities found in each examination.^5^ These videos were chosen because they were created for medical students new to the field of otolaryngology and gave a brief but comprehensive view of the major components of the physical examination that extend beyond the exclusivity of otolaryngology-interested students.

Also prior to each meeting session, students were required to read the relevant portions of the AAO-HNSF handbook, as well as supplemental learning materials specific to the various didactic sessions. Video conference sessions included lectures, case-based learning, and walk-throughs of surgical videos on the following topics: rhinology, otology, facial plastic and reconstructive surgery, laryngology, pediatric otolaryngology, head and neck imaging, and otolaryngology emergencies. The general day-to-day schedule consisted of 2 hours of morning lecture followed by a lunch break and 2 hours of afternoon lecture. Students were also provided with the option to partake in the CORONA lecture series offered by the University of Kentucky, with time reserved in their schedule to do so.^6^

To avoid “Zoom fatigue,” lecture blocks did not exceed 2 hours, and each presentation placed an emphasis on student engagement.^7^ We additionally encouraged student participation by mandating that the students’ video cameras be kept on throughout the lectures. Each day the lectures were archived for viewership among our students and any other student of the Emory medical community.

Additionally, students were required to virtually attend Emory Otolaryngology Grand Rounds on the fourth day of the course. The start of the week included an orientation to the field, and the end included a roundtable conversation where students were encouraged to ask questions about life as a resident and attending otolaryngologist.

### Evaluation

This study was determined to meet the criteria for exemption by the Emory University Institutional Review Board.

A mixed methods approach was used to collect quantitative data (via a multiple-choice question [MCQ] quiz) and qualitative data (via surveys).

#### Participant Description

The curriculum was implemented 3 out of 4 weeks from April 20 to May 15, 2020. A total of 12 participants, all rising third-year medical students, completed the course.

#### Evaluation of Student Performance

Student assessment consisted of a pre- and posttest of 12 MCQs. The pretest was administered the day before the start of the course, and students could not view the correct answers after completion. The posttest contained the same questions and was graded on a pass/fail basis, with review of the correct answers with an attending faculty member after completion. Students also completed short MCQ quizzes prior to each day of the course on the required reading for that day to assess comprehension of the reading. These quizzes were graded only for completion. On the final day, students each delivered a 5-minute presentation on an otolaryngology topic of their choice, which was also graded only for completion.

### Data Collection

Student course feedback was obtained through a postcourse survey administered on the final day. This voluntary anonymous survey was collected with Google Forms. We collected baseline demographic information about the students, including year of training, baseline levels of interest in otolaryngology and surgical fields in general, and baseline level of understanding of otolaryngology, as subjectively compared with peers in their class. Students were then asked whether the course increased these interests and whether it improved their understanding of otolaryngology. Survey responses were formatted in a 4-point Likert scale ranging from “uninterested” to “very interested,”“poor” to “very good,” and “decreased interest” to “greatly increased interest,” respectively, for the aforementioned points. Satisfaction with the course was also assessed, and if students had already completed the required third-year rotations, they were asked to remark on whether the course would have added value to the surgical clerkship. Free-text boxes were included at the end of the survey for students to discuss aspects of the course that went well and areas for improvement; prompts included the following:

Question 1: “What do you think went well with this course? Please comment specifically on content that was valuable and content that lent itself well to this format.”
Question 2: “What do you think could be improved about this course?”
Question 3: “Please provide constructive feedback about your instructors, including both strengths and weaknesses.”

### Data Analysis

#### Quantitative Analysis of Pre- vs Posttest

The pretest was not created until week 2 of the course, so the 7 students from the first week did not receive the pretest. Their posttest data were excluded from quantitative analysis.

A paired Student’s *t* test was used to compare pre- and posttest scores for the 5 students who had taken both tests.

#### Qualitative Analysis of Survey

All free-text survey responses were compiled into a single document. In a modified content analysis of the free-text responses, 2 study members (A.J.S. and M.B.S.) independently coded each document to parse the responses into discrete thematic pieces. Each response had the potential to contain multiple codes, including multiple iterations of the same code. A third study member (B.P.S.) acted as an arbiter and resolved discrepancies between the initial coded documents. The resulting final list of codes was analyzed for general concepts, and the most frequent codes in each category were noted.

## Results

### Study Participants

All 12 students were third-year medical students: 7 men and 5 women. Prior to the course, 3 students marked interest in applying into otolaryngology for residency, 8 undecided, and 1 orthopedic surgery. All students took the course for academic credit pass/fail.

### Pre- and Posttests

**Figure 1.**
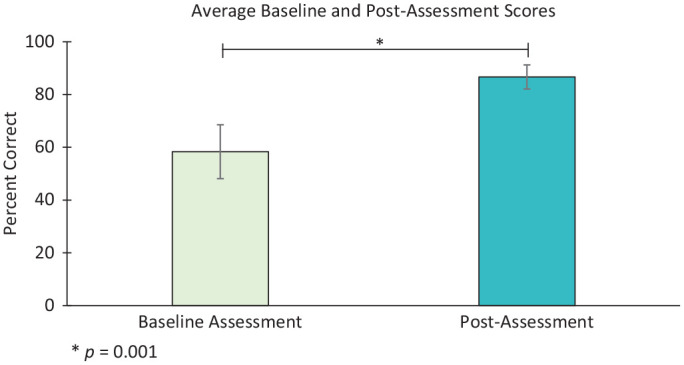
Baseline assessment (pretest) and posttest scores. Mean (SD) scores for the baseline and postcourse assessments in percentage correct were 58.3 (10.2) and 86.7 (4.6), respectively, with a mean increase in 28.3%. A paired Student’s *t* test was used to compare pre- and posttest scores for the 5 students who had taken both tests (*P* = .001). Error bars represent standard deviation.

For the 5 students with pre- and posttests, mean (SD) scores for the baseline and postcourse assessments in percentage correct were 58.3 (10.2) and 86.7 (4.6), respectively, with a mean increase in 28.3% (see **Figure 1**).

A paired Student’s *t* test revealed a significant difference between pre- and posttests (*P* = .001).

### Survey Results

The majority (92%) reported that they were “comfortable” or “very comfortable” with using online video conference software as the delivery method for the course. All students indicated that the course met the specific learning objectives in the “well–very well” range.

According to the postcourse survey, 67% of students self-assessed their baseline precourse understanding of the field of otolaryngology as compared with their peers to be in the “poor-fair” range, with the remaining students reporting a “good” understanding (n = 12). The students then self-assessed their postcourse understanding versus their peers, with 42% and 58% indicating “good” or “very good” understanding, respectively. Out of 12 students, 92% reported an increase in understanding while 1 cited no change in understanding (ie, from “good” to “good”). Furthermore, 92% of students reported either “increased” or “greatly increased” interest in the field of otolaryngology versus their baseline level of interest after taking the course (see **Table 1**). One student labeled baseline interest in otolaryngology as “indifferent” and cited “no change” in interest after the course; this student also marked orthopedic surgery as a primary interest for residency application.

**Table 1.** Postcourse Change in Interest in the Field of Otolaryngology.

Change in interest after course	Students, No. (%)
Decreased interest	0 (0)
No change	1 (8)
Increased interest	6 (50)
Greatly increased interest	5 (42)

### Analysis of Free-Text Survey Responses

The mean response rate across all 3 relevant free-response questions was 72% (n = 12). For the complete breakdown of code frequencies of questions 1 and 2, see **Table 2**.

**Table 2.** Frequency of Codes From the Free-Text Response Section of Postcourse Survey.

	Free-response question	
Frequency of codes	1: Course-positive feedback	2: Course-constructive feedback
4	Course well organized	—
3	Curriculum: enjoyed assessments Curriculum: enjoyed case-based learning	Troubleshoot technical difficulties with video streaming
2	Curriculum: enjoyed textbook Appreciated participation Breadth of topics Small group size Face-to-face faculty time	Decrease lecture time
1	Curriculum: enjoyed OR videos	Include more OR videos Maintain continuity with a single student leader Ensure faculty familiarity with technology Improve clarity of syllabus

Abbreviation: OR, operating room.

For free-text question 1, positive course feedback centered on organization (mentioned 4 times) and specific components of the curriculum, with assessments and case-based learning each mentioned 3 times.

Question 2 provided a breakdown of constructive feedback for future implementation. The most common concerns related to technical difficulties that were encountered with the video streaming service (3 comments), followed by concerns relating to the length of some of the lectures (2 comments). Further feedback suggested including more operating room surgical videos, consolidation of course leadership, and some minor suggestions about course organization.

Question 3 had the aim of gathering feedback about the performance of the instructors themselves rather than the content of the course. The overarching theme of the positive feedback received was that students appreciated that instructors were engaging and encouraged participation (4 comments). There was no clear trend for constructive feedback for instructors; there was a single mention for each of the following points: “improve organization,”“increase interactivity,” and “decrease esoteric lectures.”

## Discussion

The COVID-19 pandemic continues to affect medical student education. Social distancing measures have limited third- and fourth-year clinical experiences.^8^ Many efforts have been made to supplement educational experiences for otolaryngology residents, but little has been published on otolaryngology education for medical students.^9-12^ In our study, we focused on the implementation of a 1-week virtually taught otolaryngology elective designed for medical students. Our results included quantitative and qualitative analysis pooled from pre- and posttests and postcourse surveys. Our findings, in this admittedly small study, indicate that otolaryngology topics and interest can be taught and generated through a virtual medium.

The results of the postcourse survey indicate that students felt that they had an improved understanding of otolaryngology topics and that the learning objectives were met through the 1-week elective. After 5 days, students showed improved understanding of the material, as seen by the marked improvement on posttest scores. One MCQ was consistently answered incorrectly by students on pre- and posttest, suggesting poor quality or clarity of the question, incomplete lecture coverage of the topic, or inappropriate level of difficulty for assessment of third-year medical students. Of note, only 5 students of the 12 were able to complete a pre- and posttest. Beyond mastering the educational material, students indicated an elevated interest in the field of otolaryngology.

Course feedback provided valuable insight into the implementation of this 1-week virtual learning experience. Students liked the organization of the course, use of assessments, course textbook, and use of surgery videos. Students also liked the level of participation, small class size, enhanced face-to-face faculty opportunities, and exposure to the breadth of the field. Moving forward with the course, faculty interaction could be maintained through monthly information sessions, attendance at grand rounds presentations, and even virtual “happy hours.”

A majority of students stated that they were comfortable using the virtual medium, which, in tandem with the successes of the curriculum at meeting its stated objectives, suggests that the virtual medium is a practical and productive teaching modality. Our lectures were archived in a shared webpage available to all Emory medical students, and we intend to direct future students interested in otolaryngology learning material to this webpage and provide them with a curriculum to guide their viewing of the content.

Course feedback indicated some limitations of the virtual learning experience. Technical difficulties with video streaming and long lecture time were most frequently cited, including internet connectivity problems and lecturer difficulty with the online interface. It was also noted that, unlike in-person learning experiences, our elective relied on constant video streaming, leaving our students vulnerable to “Zoom fatigue.”^7^ Future iterations of this course could address these issues by ensuring that lecturers have appropriate training in videoconferencing technology and that students have enough break time between lectures for self-study. Student-faculty interaction during lectures could be enhanced through utilization of an MCQ polling system (eg, Poll Everywhere). This would allow students to gauge their understanding independently, compare understanding with their peers, and facilitate opportunities for students to initiate discussion with faculty.

There were additional notable limitations to this study. First, the virtual curriculum was developed in only 4 weeks to prevent gaps in student education caused by the sudden cancelation of clinical activities. Therefore, the course could not be pilot-tested prior to implementation, resulting in technical difficulties that could have been avoided. Short preparation time for the course also presented the challenge of developing a comprehensive pre- and postassessment. Utilizing the same questions on the pre- and postassessment solidified core knowledge with repetition but limited our ability to assess the breadth of information taught during the course. Furthermore, the study was limited by the small number of students who participated in the course (N = 12) and the small number of students who participated in pre- and postassessments (n = 5), as well as the inability of the sole student in the third week of the course to remain anonymous when responding to the survey.

Overall, this study shows that a 1-week otolaryngology elective provided through a virtual format can be effective at providing an educational experience and garnering interest in the field. A multifaceted curriculum with virtual learning serves as a useful adjunct to medical student education that should be continued once medical students resume clinical duties. Future directions for this course include broadening the curriculum to include telemedicine and patient interviews, as well as individualizing the curriculum for students who have already completed the third-year surgical clerkship to challenge them to perform at the level of a junior intern.

## Conclusion

We successfully introduced a foundational knowledge of otolaryngology in a remote 1-week learning environment, which met its learning objectives through interactive lectures, case-based learning, course assessments, and survey feedback. Students who took our elective demonstrated an overall positive affirmation of the virtual learning experience. They also demonstrated enhanced knowledge in otolaryngology and developed an increased interest in the specialty. Our experience suggests that virtual curricula can be utilized to enhance surgical education of medical students even after the resumption of clinical duties, especially for surgical specialties that would otherwise receive little attention.
